# Time to lowest postoperative carcinoembryonic antigen level is predictive on survival outcome in rectal cancer

**DOI:** 10.1038/srep34131

**Published:** 2016-09-23

**Authors:** Huichuan Yu, Yanxin Luo, Xiaolin Wang, Liangliang Bai, Pinzhu Huang, Lei Wang, Meijin Huang, Yanhong Deng, Jianping Wang

**Affiliations:** 1 Guangdong Provincial Key Laboratory of Colorectal and Pelvic Floor Disease, The Sixth Affiliated Hospital (Guangdong Gastrointestinal and Anal Hospital), Sun Yat-sen University, Guangzhou, Guangdong, 510655, China; 2 Department of Colon and Rectum Surgery, The Sixth Affiliated Hospital (Guangdong Gastrointestinal and Anal Hospital), Sun Yat-sen University, Guangzhou, Guangdong, 510655, China; 3 Department of Medical Oncology, The Sixth Affiliated Hospital (Guangdong Gastrointestinal and Anal Hospital), Sun Yat-sen University, Guangzhou, Guangdong, 510655, China

## Abstract

This study was to investigate whether the time to the lowest postoperative CEA can predict cancer survival. We enrolled 155 rectal cancer patients in this retrospective and longitudinal cohort study. Deepness of response (DpR) of CEA refers to the relative change of the lowest postoperative CEA level from baseline, and time to DpR (TTDpR) refers to the time from surgery to the lowest postoperative CEA level. The median of TTDpR and DpR was 4.5 (range, 3.0–18.0) weeks and −67% (range, −99% to 114%) respectively. Patients with TTDpR </ = 4.5 weeks had better 3-year DFS (81.4% vs. 76.2%; P = 0.059) and OS (95.8% vs. 87.9%; P = 0.047) rate than patients with TTDpR >4.5 weeks. Using TTDpR as a continuous variable, the HR of DFS and OS was 1.13 (95% CI 1.06–1.22, P = 0.001) and 1.17 (95% CI 1.07–1.29, P = 0.001) respectively. On multivariate analysis, the predictive value of prolonged TTDpR remained [adjusted HRs: 1.12 (95% CI 1.03–1.21, P = 0.006) and 1.17 (95% CI 1.06–1.28, P = 0.001)]. These findings remained significant in patients with normal preoperative CEA. Our results showed prolonged TTDpR of CEA independently predicted unfavorable survival outcomes, regardless of whether preoperative CEA was elevated or not.

Although the degree of penetration of the primary lesion (T stage) and nodal status (N stage) provides the best prognostic information about survival and disease relapse after surgery alone or combined with adjuvant treatment for colorectal cancer (CRC)^1^, some patients with the same stage would have different prognosis. The challenge lies in identifying the risk factors that type the subsets at high risk for recurrence in rectal cancer. It is now clearly established that patients with elevated preoperative carcinoembryonic antigen (CEA) levels have poorer survival outcome^2^,^3^,^4^,^5^. However, some controversies have existed concerning the significance of the preoperative CEA level as an independently predictive factor of recurrence and survival^6^. Some studies have shown the preoperative CEA level to be an independent predictor for DFS only in patients with TNM stage III^7^,^8^ or stage II^7^,^9^. Other pretreatment markers, such as Modified Glasgow Prognostic Score^10^, C-reactive protein/albumin ratio^11^ and neutrophil to lymphocyte ratio^12^, have been identified as predictors of an unfavorable outcome following surgical resection of rectal cancer. However, the validity of those markers remains controversial, and their clinical application is limited because of their limited predictive value and high cost.

In the absence of preoperative predictors of survival, early on-treatment changes may help identify those patients in whom tumor resection and combined treatments are worthwhile. Perioperative change of CEA is an example of such a parameter. Patients with continuously elevated CEA in both preoperative and postoperative period are more likely to develop systemic recurrence and cancer-related death in rectal cancer patients^13^,^14^,^15^. Unfortunately, the prognosis of the patients with normal CEA level in both periods or patients with preoperatively elevated and postoperatively normal CEA still varies widely. Recent studies have shown strong correlations between early tumor shrinkage and response to cetuximab in metastatic colorectal cancer treatment (mCRC)^16^. Specifically, data from large prospective randomized trials such as FIRE-3, CRYSTAL, TRIBE and PEAK showed deepness of response (DpR) in tumor size consistently predicted overall survival (OS) in clinical trials^17^,^18^,^19^,^20^.

From these data, we hypothesize that DpR in serum CEA and time to DpR (TTDpR), irrespective of whether the preoperative CEA is elevated or not, are markers of treatment effect and could be used to predict long-term survival in rectal cancer treated with radical surgery. The main purpose of this study was to validate the predictive value of DpR and TTDpR of CEA on disease-free survival (DFS) and OS in an independent series. For this, we conducted a retrospective analysis on a landmark trial of rectal cancer.

## Methods

### Patients

**Figure 1:**
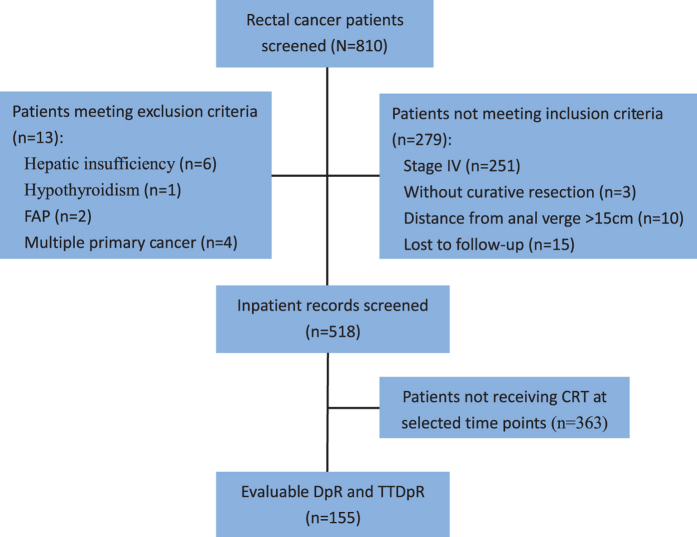
Patient disposition in the analysis of the effect of DpR and TTDpR on survival in rectal cancer.

This is a retrospective and longitudinal cohort study of stage I to III rectal cancer patients who underwent a curative resection with adjuvant chemoradiotherapy (CRT) in our institution from June 2007 and June 2011. Rectal cancer was defined as histologically proven adenocarcinoma within 15 cm from the anal verge and was staged according to the 7th edition of the American Joint Committee on Cancer (AJCC) staging system^21^. The patients were included in the cohort if they received adjuvant CRT three to five weeks after the surgery for six to eight 21-day cycles with serum CEA sampling before each cycle of CRT was given. Because frequent sampling is very difficult in postoperative patients, the evaluation of TTDpR of CEA was feasible only in these selected patients. Patients with hepatic insufficiency, end-stage lung diseases, hypothyroidism, familial adenomatous polyposis (FAP) and multiple primary cancer were excluded (Fig. 1). Staging procedures, including colonoscopy, contrast-enhanced CT scans of the thorax, abdomen and pelvis and pelvic MRIs, were performed in all cases to exclude patients with evidence of distant metastatic disease at the initial diagnosis. The data regarding demographic characteristics (age, height, and weight), history of smoking, tumor location, tumor staging, tumor’s histological features, presence or absence of blood transfusion, perioperative serum carcinoembryonic antigen (CEA) level, treatment regimen and time to recurrence and survival were collected from the Institutional Cancer Database and inpatient records.

### Treatment

The detail of CRT in the protocol was described in our previous publication^22^. Forty-eight out of 155 patients (31%) received neoadjuvant CRT, in which a total irradiation dose of 46 Gy in 23 fractions of 2 Gy with concomitant application of 5-fluorouracil (5-FU) was given. At a median interval of 10.9 weeks (range, 6.9–17.3) after completion of CRT, total mesorectal excision (TME) for rectal cancer was implemented. Surgical approaches comprised low anterior resections, abdominoperineal resections and Parks procedure. Perioperative assessment of TME quality was performed in all surgical specimens. According to physicians’ suggestions and patients’ decisions, postoperative 5-FU based chemotherapy was implemented following surgery for six to eight cycles with a median interval of 4.3 (range, 3.0–5.0) weeks.

### Carcinoembryonic antigen assessment

**Figure 2:**
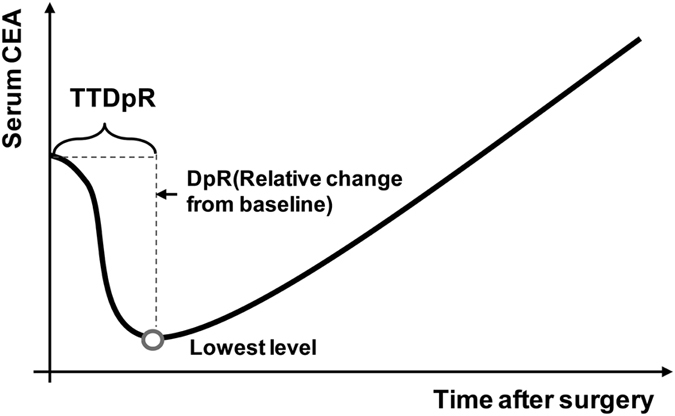
The curve of CEA change in most patients treated with adjuvant chemotherapy.

Serum CEA levels were measured at baseline (before surgery) and each cycle of adjuvant CRT (week 3–5, 6–8, 9–11, etc.) until the end of adjuvant CRT. The relative change of CEA from baseline was computed at each time point. In this study, two novel methods were proposed. Deepness of response of serum CEA (DpR of CEA) refers to the relative change of lowest postoperative CEA level from baseline, and time to DpR of CEA (TTDpR of CEA) refers to the time to the lowest postoperative CEA level (Fig. 2). All of preoperative CEA, DpR and TTDpR were used to evaluate the predictive effect on survival outcome. The normal limit of serum CEA measured by RIA was set as <5 ng/mL in our institution. TTDpR was evaluated as both continuous and categorical variables using the median value.

### Follow-up

Patients were followed up every three months for the first two years and every six months thereafter. Each visit consisted of pertinent medical history, physical examination, including rectal examination, and measurement of serum CEA levels. Colonoscopy and radiological examinations consisting of chest radiography, abdominopelvic CT and ultrasonography were scheduled every six months for the first three years and annually thereafter. The follow-up period for the study ended July 2014 with the interval of follow-up varying from three to seven years. Cancer recurrence was detected by CEA >5 ng/mL and/or a sequential computerized tomography scan with evidence of the disease followed by histopathological confirmation. The primary endpoints were disease-free survival (DFS) and overall survival (OS). DFS was defined as the time from the surgery until recurrence or death from any cause, and OS was defined as the time from the surgery to death.

### Statistical analyses

Data analyses were performed using SPSS version 19.0 for Windows (SPSS, Inc., Chicago, IL). The intergroup comparisons of clinicopathologic variables were performed by variance and Kruskal–Wallis tests for continuous variables (depending on the distribution of the continuous variables), and the chi-square and two-tailed Fisher’s exact tests for discrete variables. The overall survival (OS) rate and disease-free survival (DFS) rate were estimated and compared according to the Kaplan-Meier method and log-rank test, respectively. A univariate screen of all potential predictors of DFS and OS using the Cox proportional hazard model for all the collected clinicopathological data was performed. All the variables appear to be significantly associated with survival were included in the multivariate Cox’s proportional hazard model with backward Wald stepwise elimination procedure to identify the independent risk factors. Subgroup analysis for the association of TTDpR with survival was performed by preoperative CEA level (normal or elevated). A p-values <0.05 (two-sided test) was considered statistically significant. The study was powered to evaluate the survival rate with an estimate hazard ratio (HR) of 1.2. A minimum of 147 patients was required to detect the aforementioned estimated difference in survival with 80% power and <5% type 1 error^23^,^24^.

### Ethics Statement

The study was approved by the Medicine Ethics Committee of the Hospital in Sun Yat-sen University. There was no harm to patients, given that the data were collected retrospectively from database and medical records. All the necessary precautions were taken to secure the privacy of the human subjects in our database, allowing the medical records and databases to be used only by the investigators. The human subject protocol was approved by the Committee on Clinical Investigation. Informed consent was obtained from all human subjects in accordance with The World Medical Association Declaration of Helsinki. All the methods were also carried out in accordance with the approved guidelines (http://jama.jamanetwork.com/article.aspx? articleid=1760318).

## Results

### Baseline clinicopathologic features

A total of 155 patients matched the inclusion and exclusion criteria and were included in this study (Fig. 1). The clinicopathologic features of the each group categorized by TTDpR were summarized in Table 1. The median age of these patients was 55 years. There were no significant differences among the groups with regard to age, sex, BMI, smoking status, deepness of infiltration, lymph node status, grade of differentiation, vascular invasion, perineural invasion and tumor location, whereas the patients with TTDpR > 4.5 weeks had significantly higher incidence of preoperative elevated CEA than those with TTDpR </ = 4.5 weeks (33% vs. 19%; P = 0.038).

The preoperative CEA level was distributed with a median of 2.25 (range, 0.34–118.00) ng/ml, and 39 (26%) patients had elevated preoperative CEA, among which elevated CEA was normalized three to five weeks following surgery in 37 (95%) patients. Interestingly, regardless of the preoperative CEA level, 152 of 155 patients receiving adjuvant CRT had CEA initially decreased after tumor resection and then raised (Fig. 2), and the other three patients had an increasing postoperative CEA compared with the baseline, as shown in change of tumor size in metastatic CRC treatment. The median of TTDpR was 4.5 (range, 3.0–18.0) weeks, and the median of DpR was −67% (range, −99% to 114%).

### Survival analysis

**Figure 3:**
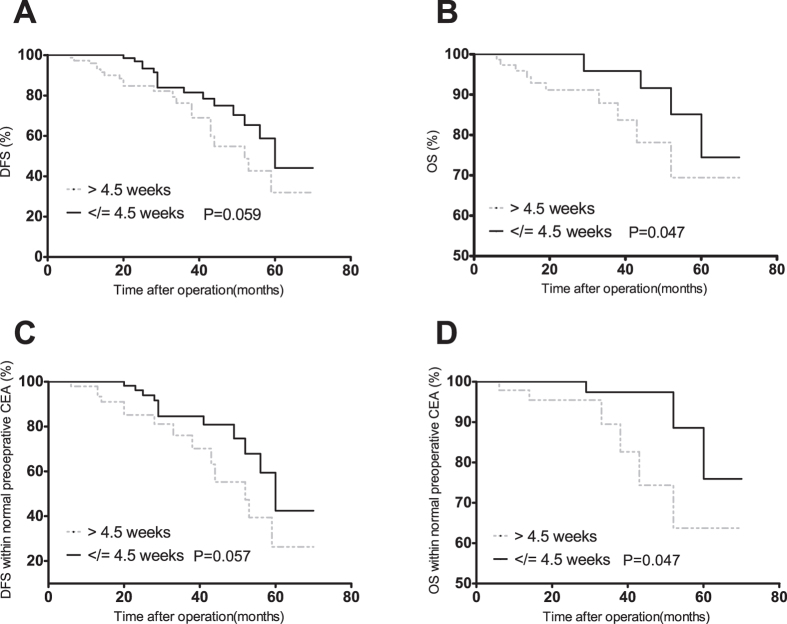
The Kaplan–Meier curves of DFS (**A**) and OS (**B**) for different TTDpR in all the patients. The Kaplan–Meier curves of DFS (**C**) and OS (**D**) for different TTDpR in the patients with normal preoperative CEA level.

The univariate analysis indicated that advanced AJCC stages, low-grade differentiation, vascular invasion, perineural invasion and low rectal cancer were significantly associated with worse 3-year DFS and OS outcome. Preoperative elevated CEA level was associated with low 3-year DFS (65.3% vs. 75.6%, P = 0.024) and OS (71.2% vs 87.5%, P = 0.004) rate, the HRs of which were 1.46 (95% CI 1.01–2.09, P = 0.044) and 2.22 (95% CI 1.40–3.50, P = 0.001) respectively. (Table 2) When TTDpR of CEA was evaluated as a continuous variable, the HRs of DFS and OS rate were 1.13 (95% CI 1.06–1.22, P = 0.001) and 1.17 (95% CI 1.07–1.29, P = 0.001) respectively. We also observed that patients with TTDpR </ = 4.5 weeks had higher 3-year DFS (81.4% vs. 76.2%; P = 0.059) and OS (95.8% vs. 87.9%; P = 0.047) rate than patients with TTDpR >4.5 weeks, the HRs of which were 0.55 (95% CI 0.29–1.04, P = 0.065) and 0.35 (95% CI 0.12–0.97, P = 0.048) respectively (Table 2, Fig. 3A,B). However, Kruskal–Wallis tests showed DpR had no predictive value on either DFS or OS.

Multivariate Cox analysis showed that advanced AJCC stage, perineural invasion and prolonged TTDpR [adjusted HR 1.12 (95% CI 1.03–1.21), P = 0.006] were independently associated with DFS. Another multivariate Cox regression analysis looked at the independent factors found OS was determined by prolonged TTDpR [adjusted HR 1.17 (95% CI 1.06–1.28), P = 0.001], advanced AJCC stage and low rectal cancer (Table 3).

### Additional Analyses in patients with normal preoperative CEA

Further subsets analyses were conducted to determine if the above predictive effect of TTDpR on survival was affected by preoperative CEA or merely depends on normalization of elevated CEA. After excluding 39 (25%) patients with elevated preoperative CEA level, 116 patients were analyzed. The Kaplan–Meier curves showed both the three-year DFS (76.0% vs. 84.6%, P = 0.057) and OS (89.5% vs. 97.4%, P = 0.047) rate were lower in patients with TTDpR >4.5 weeks in comparison to patients with TTDpR </ = 4.5 weeks (Fig. 3C,D). When the multivariate Cox regression analyses were carried out, we found prolonged TTDpR remaining an independent predictor of DFS with adjusted HR equal to 1.21 (95% CI 1.04–1.40, p = 0.012), while OS was not determined by TTDpR in the analyses.

## Discussion

The major findings of this study were that rectal cancer patients with less TTDpR showed better survival rates than those with prolonged TTDpR, which was still an independent predicting factor in patients with normal preoperative CEA level. TTDpR has potential to be a reliable predictor of long-term survival. As far as we are aware, this is the first time to propose the concept of TTDpR of CEA and report on its association with cancer outcomes. In addition, we also confirmed and built on the findings in reports by other authors^9^,^25^,^26^, where preoperative CEA level was associated with the long-term survival.

It may be postulated that the association of time to the lowest postoperative CEA with survival outcomes is more likely to depend on the normalization of CEA in the 39 patients with elevated preoperative CEA. However, the fact that the significant predictive value of less TTDpR on better DFS and OS remained after excluding these 39 patients suggests that these findings were not be attributed solely to the normalization of CEA. Therefore, it is likely that the TTDpR per se is truly related to cancer survival, irrespective of whether the preoperative CEA is elevated or not. According to those results, the predictive effect applies to all included 155 patients.

Serum CEA is the most widely accepted tumor marker for colorectal cancer due to its high expression in adenocarcinomas and its standardized, readily available and cost-effectively measurement. However, it is also expressed in normal mucosal cells^27^. The American Society of Clinical Oncology Tumor Marker Expert Panel has recommended preoperative and postoperative measurement of CEA levels every 3 months for stage II and III disease for at least 3 years^28^. Its most useful role in surveillance is after R0 resection in colorectal cancer, but has limitations as a marker of recurrence, as elevations of CEA can be seen in several non-neoplastic conditions, as active inflammation of the colon and rectum, renal or hepatic insufficiency, end-stage lung diseases, hypothyroidism, obesity, aging, and cigarette smoking^29^,^30^,^31^,^32^,^33^. Overall, there is a false positive rate of 7 to 16%^34^. In our series, we have controlled these factors that influence differential expression of CEA.

Not unexpectedly, we validated elevated preoperative CEA level predicts long-term survival, which was consistent with previous results of most studies^9^,^25^,^26^,^35^. Although the association was significant, patients with normal preoperative CEA level merely had an 11% higher rate of 3-year DFS than patients with elevated preoperative CEA level (76.5% vs. 65.3%), which suggested high heterogeneity within groups.

It has been postulated that surgery may encourage both the implantation of surgically disseminated tumor cells and the growth of existing micrometastases, and perioperative modulation of immunocompetence might have significant effects on oncological outcomes^36^. Hence it is essential to identify biomarkers not merely reflecting the biological behavior of the tumors but the anti-cancer outcome of tumor resection combined with medical treatment and host immune defense over prolonged periods of time. Hypothesizing that CEA reflects tumor burden, including resected primary tumor bulk and residual tumor cells, the DpR and TTDpR of CEA might correlate with the amount of tumor elimination and thus long-term outcome. Some studies reported the constantly elevated perioperative CEA predicts bad prognosis^13^,^14^,^15^, however, it is confined to patients with elevated both preoperative and postoperative CEA whose incidence is low in the population. Therefore, DpR and TTDpR of CEA after curative resection were proposed to be evaluated. The fact that the significant association between TTDpR and survival outcomes remained in the subsets with normal preoperative CEA suggests that TTDpR had the potential to be such a surrogate marker, although it is based on a collective of patients receiving adjuvant CRT in a single center. Above all, it will be of great significance to compare the relative change of other biomarkers to identify their DpR and TTDpR as novel and valuable predictors of survival outcomes.

The CEA wash-out phase approximated four weeks^37^ and the median of TTDpR was coincidently equal to 4.5 weeks in our series, by which patients were categorized into two groups. Consequently, we found patients with time to lowest postoperative CEA level less than 4.5 weeks had better survival outcomes than that with the time more than 4.5 weeks. The difference remained significant in the subsets with normal preoperative CEA. These findings may be attributed to the CEA wash-out phase and predicting value of TTDpR.

Nevertheless, this study has some limitations, including a small number of patients for analyses of TTDpR and the single-institution design. The measurement of CEA varies by institution, thus the data from other institutions or a multi-center study design are essential to validate those results. Then, as for the limitation of sample size, we couldn’t provide subgroup analysis for neoadjuvant CRT (only 48 patients). It is necessary to determine if TTDpR functions best in patients with/without neoadjuvant CRT in future larger cohort. Moreover, we cannot rule out selection bias in our retrospective case series, in which many patients were excluded due to absence of serum CEA levels or due to different protocol of adjuvant treatment. But above all, studies prospectively and more frequently collecting CEA at each predetermined time point will be necessary to validate the association and obtain an optimal cut-off value of TTDpR of CEA. Since that frequent CEA sampling after surgery is difficult to be acceptable, the data from the present retrospective study would provide the evidence for a prospectively designed study.

In conclusion, TTDpR is a novel marker to predict survival outcome in rectal cancer, which might be associated with predictive value of anti-cancer outcome of surgery combined with medical treatment and host immune defense. Our results showed that less TTDpR was an independently predictor of better DFS and OS, regardless of whether preoperative CEA was elevated or not. TTDpR had potential to be an early-stage marker of efficacy in clinical treatment, as such or in combination. However, larger prospective studies will be needed to validate the association and the cut-off value. Furthermore, it will be useful to investigate of the relative change of other known biomarkers to develop their DpR and TTDpR as a novel and valuable predictors.

## Additional Information

**How to cite this article**: Yu, H. *et al*. Time to lowest postoperative carcinoembryonic antigen level is predictive on survival outcome in rectal cancer. *Sci. Rep.* **6**, 34131; doi: 10.1038/srep34131 (2016).

## Figures and Tables

**Table 1:** Clinicopathologic features in patients with rectal cancer according to TTDpR.

Characteristic	Overall population (N = 155)	TTDpR		
		>4.5 weeks (N = 75)	</ = 4.5 weeks (N = 80)	p-value
Age-yr, median (range)	55 (21–78)	56 (21–75)	54 (25–78)	0.242
BMI, median (range)	22.05 (14.22–33.79)	21.80 (15.63–33.79)	22.39 (14.22–30.86)	0.457
Sex, no.(%)				0.416
Male	92 (59)	47 (63)	45 (56)	
Female	63 (41)	28 (37)	35 (44)	
Smoking history, no.(%)				0.548
No	121 (78)	57 (80)	64 (78)	
Yes	34 (22)	18 (20)	16 (22)	
Tumor category, no.(%)				0.849
T1	4 (3)	2 (3)	2 (2)	
T2	25 (16)	12 (16)	13 (16)	
T3	113 (73)	55 (73)	58 (73)	
T4	13 (8)	6 (8)	7 (9)	
Nodal category, no.(%)				0.206
N0	61 (39)	33 (44)	28 (35)	
N1-2	94 (61)	42 (56)	52 (65)	
TNM Stage, AJCC, no.(%)				0.512
I	19 (12)	10 (13)	9 (11)	
II	42 (27)	23 (31)	19 (24)	
III	94 (61)	42 (56)	52 (65)	
Differentiation degrade, no.(%)				0.450
Low	40 (26)	20 (27)	20 (25)	
Moderate	76 (50)	39 (54)	37 (47)	
High	36 (24)	14 (19)	22 (28)	
Lymphovascular invasion, no.(%)				0.310
Positive	21 (13)	67 (89)	67 (84)	
Negative	134 (87)	8 (11)	13 (16)	
Neural invasion, no.(%)				0.298
Positive	26 (17)	15 (20)	11 (14)	
Negative	129 (83)	60 (80)	69 (86)	
Preoperative CEA				0.038
Normal	115 (74)	50 (67)	65 (81)	
Elevated	40 (26)	25 (33)	15 (19)	
Distance from anal verge, no. (%)				0.474
<5 cm	58 (38)	30 (41)	28 (35)	
5–12 cm	94 (62)	43 (59)	51 (65)	
Unknown	3	0	3	
Surgical approach, no.(%)				0.245
Low anterior resection	114 (73)	51 (68)	63 (79)	
Abdminoperineal resection	15 (10)	10 (13)	5 (6)	
Parks procedure	26 (17)	14 (19)	12 (15)	
Neoadjuvant treatment, no.(%)				0.507
No	107 (69)	52 (71)	53 (66)	
Yes	48 (31)	21 (29)	27 (34)	

**Table 2:** Univariate analysis of risk factors for three-year DFS and OS.

Risk Factor	Disease-free Survival			Overall Survival		
	3-year DFS rate (%)	HR (95% CI)	p-value	3-year OS rate (%)	HR (95% CI)	p-value
AJCC Stage III	62.2	4.06 (2.27–7.23)	<0.001	75.8	5.19 (2.23–12.10)	<0.001
AJCC Stage II	78.7	2.05 (1.08–3.89)	0.027	82.3	3.17 (1.29–7.74)	0.012
Low-grade differentiation	61.4	2.32 (1.37–3.93)	0.002	72.5	4.28 (1.93–9.50)	<0.001
Vascular invsion	53.1	2.32 (1.50–3.58)	<0.001	67.3	2.30 (1.31–4.03)	0.004
Perineural invasion	37.8	3.60 (2.27–5.69)	<0.001	70.0	2.41 (1.27–4.59)	0.007
Distance from anal verge <5 cm	69.4	1.50 (1.06–2.13)	0.022	77.1	1.58 (1.02–2.47)	0.043
Preoperative elevated CEA	65.3	1.46 (1.01–2.09)	0.044	71.2	2.22 (1.40–3.50)	0.001
TTDpR		1.13 (1.06–1.22)	0.001		1.17 (1.07–1.29)	0.001
</ = 4.5 weeks	81.4	0.55 (0.29–1.04)	0.065	95.8	0.35 (0.12–0.97)	0.048
>4.5 weeks	76.2	1		87.9	1	

**Table 3:** Multivariate analysis of risk factors for three-year DFS.

Risk Factor	HR (95% CI)	p-value
Disease-free survival		
AJCC Stage III	3.94 (1.41–11.06)	0.009
Perineural invasion	3.95 (1.69–9.26)	0.002
TTDpR	1.12 (1.03–1.21)	0.006
Overall survival		
AJCC Stage III	8.50 (1.14–63.29)	0.037
Distance from anal verge <5 cm	2.62 (1.30–5.30)	0.007
TTDpR	1.17 (1.06–1.28)	0.001
